# Carbapenem-Resistant Gram-Negative Bacterial Infections at a Tertiary Health Care Center in Nepal: An Observational Study

**DOI:** 10.31729/jnma.8862

**Published:** 2025-01-31

**Authors:** Dimpi Konwar, Navin Kumar Chaudhary, Poonam Yadav

**Affiliations:** 1 Chitwan Medical College, Bharatpur, Chitwan, Nepal; 2 Department of Microbiology, Chitwan Medical College, Bharatpur, Chitwan, Nepal

**Keywords:** *antibiotic resistance*, *carbapenems*, *gram-negative bacteria*

## Abstract

**Introduction:**

Carbapenems are last-resort antibiotics and are considered the drugs of choice for infections caused by multi-drug-resistant bacteria. During the last several years, there has been an alarming global increase in the detection and spread of Carbapenem-resistant organisms among gram-negative bacteria. Therefore, the main objective of this study is to determine Carbapenem-resistant gram-negative bacterial infections.

**Methodology:**

A descriptive cross-sectional study was performed. Ethical approval was granted by the Institutional Review Committee (Reference number: CMC-IRC/080/081-071). A total of 3149 non-repeated, different clinical specimens were collected, from November 2023 to February 2024, processed aseptically under the standard protocol of the American Society for Microbiology, and screened according to the Antibiotic sensitivity pattern. The analysis of the results was performed using Microsoft Excel and manual calculations.

**Results:**

Out of 3149 samples, 361 had culture-positive. Among 361 isolates, 316 were Gram-negative bacteria, among the specimens, 83 (26.26%) were identified as Carbapenem-resistant gram negative bacteria. Within this group, *Acinetobacter baumannii* was present in 37 (44.57%) cases, followed by *Escherichia coli* with 20 (24.09%), *Klebsiella pneumoniae* with 19 (22.89%), *Stenotrophomonas maltophilia* with 3 (4.81%), and *Pseudomonas aeruginosa* and *Klebsiella aerogenes,* each with 2 (2.40%) cases. The most effective antibiotics against Carbapenem-resistant *Enterobacteriaceae* included Colistin and Fosfomycin, whereas Carbapenem-resistant non-fermenter included Colistin and Tigecycline.

**Conclusions:**

Among Carbapenem-resistant Organisms, *Acinetobacter baumannii* was most prevalent. The results revealed a significant proportion of infections resistant to commonly used antibiotics, highlighting an alarming trend in antibiotic resistance.

## INTRODUCTION

The emergence of carbapenem-resistant bacteria represents a significant public health concern worldwide. Carbapenems are among the last lines of drugs against multidrug-resistant infections, and the rise in resistance undermines their effectiveness. The World Health Organization (WHO) has highlighted this issue, categorizing carbapenem-resistant infections as a global concern that urgently requires new therapeutic approaches.^1^

A study done in Asia shows the prevalence of Carbapenem-resistant *Enterobacteriaceae* (CRE) infection ranges from 0.60-0.90%.^2^ Studies conducted in Nepal have documented resistance rates ranging from 5% to 25%,^3^"^6^ indicating a concerning trend that could complicate treatment regimens and increase healthcare costs.

The study aims to provide updated prevalence data specific to Chitwan Medical College Teaching Hospital (CMCTH), reflecting any change in resistance trend. The local data is key to understanding regional resistance patterns in Nepal, assessing infection control initiatives at CMCTH, guiding antibiotic stewardship, and informing policymakers on antibiotic resistance.

## METHODS

A hospital-based observational cross-section study was conducted at the Department of Clinical Microbiology, Chitwan Medical College Teaching Hospital (CMC-TH), from November 2023 to February 2024. Ethical approval for the study was obtained from the Institutional Review Committee (Reference number: CMC-IRC/080/081-071). All clinical specimens (Urine, sputum, endotracheal aspiration, pus, tissue, and blood) received during the study period were included. The specimens were collected from patients of any age, suspected of infection, and admitted to various wards of CMC-TH. Repeated specimens, improperly labeled, non-sterile, and wrong samples were excluded from the study.

The specimens were collected aseptically and processed by standard methods recommended by the American Society for Microbiology (ASM).^7^ There were 3,149 samples submitted for testing in the microbiology laboratory. The specimens were inoculated into appropriate media, including blood agar, Chocolate agar, Cystine Lactose Electrolyte Deficiency (CLED) agar, and MacConkey agar. Among these, 361 samples showed bacterial growth. They were further processed for identification using Gram staining to differentiate between Gram-positive and Gram-negative bacteria based on the chemical and physical properties of cell walls. Based on growth technology, the VITEK 2 (VITEK Automated Identification and Antimicrobial Susceptibility Testing System) compact automated microbiology system, was utilized to identify organisms and obtain their antibiotic sensitivity profiles.^8^ Two distinct inoculum dilutions were prepared, one was used for identification, using a suitable card based on microscopical appearance and Gram reaction, while the other was put via AT (Antimicrobial Susceptibility Testing card) cards for the Antibiotic sensitivity test (AST).

The carbapenem-resistance organisms (CRO) isolates were identified by guidelines recommended by Centres for Disease Control and Prevention (CDC), which define CRO as multidrug-resistance organisms (MDRO) that are resistant to at least one of the carbapenem antibiotics (ertapenem, meropenem, doripenem or imipenem) or produce a carbapenemase.^9, 10^

The collected data were summarized, presented, and analyzed using Microsoft Excel and manual calculations. Findings are present in frequencies and percentages.

## RESULTS

A total of 3149 samples were received for testing in the microbiology lab. Out of those, 361 (11.46%) had bacterial growth, while the rest were negative. Among the 361 positive samples, 316 (87.53%) were Gram-negative isolates. Among 361 isolates, 83 (22.99%) were identified as Carbapenem-Resistant Organisms.

Within 316 Gram-negative isolates, 83 (26.26%) were classified as CRO (Carbapenem-Resistant Organisms), and the remaining 233 (73.74%) were non-CRO.

**Figure 1:**
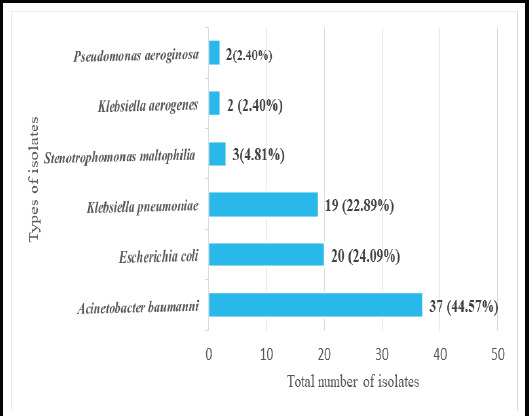
Representation of carbapenem-resistant Gram-negative bacteria (n=83).

Out of the 83 carbapenem-resistant Gram-negative bacteria (CR-GNB), 37 (44.57%) were *Acinetobacter baumannii,* (Figure 1).

**Figure 2:**
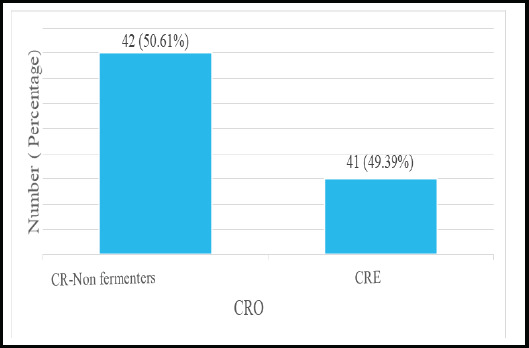
Representation of CRE and CR Non-fermenter (n=83) CRO: Carbapenem-resistance organisms; CRE: Carbapenem-resistant *Enterobacteriaceae*

Among 83 CRO isolates, carbapenem-resistant *Enterobacteriaceae* (CRE) and carbapenem-resistant non-fermenters were 41 (49.39%) and 42 (50.60%) respectively (Figure 2).

**Figure 3:**
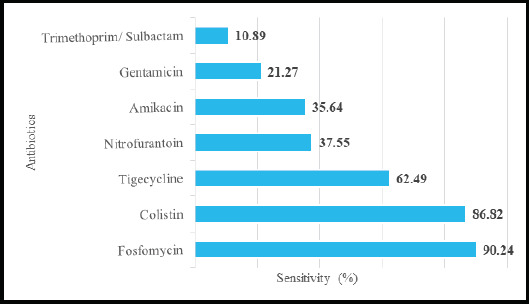
Antibiotic sensitivity percentage among Carbapenem-resistant *Enterobacteriaceae* (CRE)- infected population (n=83).

In an analysis of the sensitivity pattern, Fosfomycin was observed to be sensitive in 37 (90.33%) of cases and Trimethoprim/Sulbactam was sensitive in 4 (10.89%) of cases, (Figure 3).

**Figure 4:**
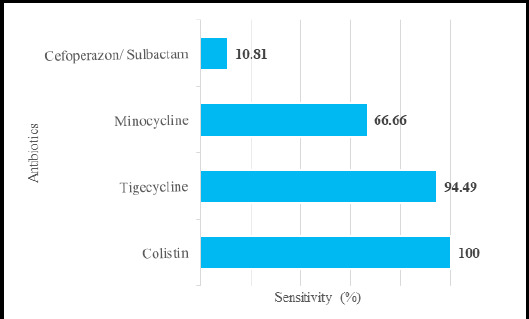
Antibiotic sensitivity percentage among CR non-fermenters-infected population (n=83).

There were 42 (100%) CR-non fermenters, and all were sensitive to Colistin, (Figure 4).

## DISCUSSION

In the present study, the prevalence of carbapenem resistance among the total culture positive was 83 (22.99%) which falls under the range of 5% to 25% which is the reported prevalence of Nepal ^3-6^ studies carried out from 2018 to 2022. Comparing these findings with previous studies, revealed that Prabhala et al. reported a similar prevalence of 26.5%, while Ansari et al. found a higher prevalence of 38.3%.^11,12^

While studying the prevalence of CRO among 316 Gram-negative isolates, it shows 83 (26.26%). In contrast, a similar study performed by Hammour et al. shows a higher prevalence of 41.2%.^13^ The difference in the resistance landscape may be due to variability in antibiotic prescribing practice, Healthcare access and quality, resource availability, the practice of prevention and control, hygiene, the environment allowing resistant organisms to spread, global trade and travel, and social determinants of health.

In the present study, the prevalence of carbapenem resistance among the bacteria belonging to *Enterobacteriaceae* was (41/230) 17.6%. In a similar study done by Karn S et al. from Nepal, and Datta et al. from India the prevalence of carbapenem resistance among the bacteria belonging to Enterobacteriaceae ranged from 4.49% to 20% and 7.87% respectively.^14-15^ Similarly, Gupta et al. also reported the prevalence of carbapenem resistance among Enterobacteriaceae to vary from 17% to 22%.^5^ In contrast, the study by Adhikari et al. conducted at Bharatpur Hospital found that the prevalence of CRE was higher at 40.7% compared to the present study.^16^ Studies have highlighted improper medication practices in Nepal, which contribute to the rise of antibiotic resistance.^17^ However, a comparison of various studies indicates the growing awareness among healthcare workers and individuals about the risks of improper drug use and self-medication, which may contribute to the rise in antibiotic resistance. The present study serves as an informative report showing the progress in addressing this issue and reducing antibiotic resistance.

In the present study, non-fermenter CRO (49.3%) had higher rates of carbapenem resistance than *Enterobactereals* (17.6%%). A similar finding was observed in a study done by Prabhala et al. whereas a study done by Saucedo et al. found the opposite result. ^11,18^ The variation may be due to the difference in sample size, study duration, and regional determinants.

In the present study, among CRO, *Acinetobacter baumanni* was most commonly isolated at 37 (44.57%) followed by *Escherichia coli* 20 (24.09%) and *Klebsiella pneumoniae* 19 (22.89%). A similar finding was reported in a study done by Hammour et al.^13^ A higher finding of *Escherichia coli* was reported by Saucedo et al.^18^ Whereas in studies done by Pokharel etal. Prabhala et al. Hammour et al. Adhikari etal. and Armin et al. reported *Klebsiella pneumonia* as the predominant CRO.^19,11,16,20^. The differences are due to sampling methods, inclusion and exclusion criteria, and regional determinants.

In the current study, antibiotic sensitivity tests on Carbapenem-resistant samples, the results showed that Amikacin, Colistin, Fosfomycin, Nitrofurantoin, Tigecycline, Gentamicin, and Trimethoprim/Sulbactam exhibited susceptibility rates of 35.64%, 86.82%, 90.33%, 37.55%, 62.49%, 21.27%, and 10.89% respectively. In comparison, the study performed by Pokharel et al. shows a similar susceptibility rate in Amikacin (33%) with a complete susceptibility rate in colistin and tigecycline and a lesser susceptibility rate in Nitrofurantoin. ^3^ On the other hand, the study by Armin et al. revealed tigecycline was the most effective antibiotic with a susceptibility rate of 62%, which aligns with our current study.^19^ Pogue et al. reported susceptibility rates of 85%, 82%, and 81% for Fosfomycin, colistin, and tigecycline respectively, similar to our results for Fosfomycin and colistin, but tigecycline showed a lower rate.^20^ Shrief et al. study demonstrated 67.4% sensitivity to colistin and 82.6% to Fosfomycin, with colistin showing a higher rate in our current study.^21^ The difference in the susceptibility rate of drugs may be due to different geographical areas, infections acquired, susceptible organisms, etc.

In the current study, Colistin showed an excellent sensitivity rate (100%) against non-fermenter CRO. A similar finding was achieved in a study done by Yadav et al. because the Antibiotic sensitivity pattern was observed in the same group of organisms with similar isolations.^22^

The study has several limitations that may impact its findings. The short study duration restricts long-term observations, while the single-center design limits the generalizability of the results. A lack of resources for further diagnosis hinders the accurate identification of conditions, and the inability to determine resistance mechanisms is a significant oversight. Additionally, not all possible risk factors were assessed, and a distinction between community-acquired and hospital-acquired infections was not made.

## CONCLUSIONS

The results revealed a significant proportion of infections resistant to commonly used antibiotics, highlighting an alarming trend in antibiotic resistance. Overall, this study provides valuable insights but emphasizes the need for more comprehensive research to address antibiotic resistance effectively.
